# Copeptin does not accurately predict disease severity in imported malaria

**DOI:** 10.1186/1475-2875-11-6

**Published:** 2012-01-05

**Authors:** Marlies E van Wolfswinkel, Dennis A Hesselink, Ewout J Hoorn, Yolanda B de Rijke, Rob Koelewijn, Jaap J van Hellemond, Perry JJ van Genderen

**Affiliations:** 1 Department of Internal Medicine, Harbour Hospital and Institute for Tropical Diseases, Haringvliet 2, 3011 TD Rotterdam, The Netherlands; 2 Department of Internal Medicine, Erasmus MC, Rotterdam, The Netherlands; 3 Department of Clinical Chemistry, Erasmus MC, Rotterdam, The Netherlands; 4 Laboratory of Parasitology, Harbour Hospital and Institute for Tropical Diseases, Rotterdam, The Netherlands; 5 Department of Medical Microbiology and Infectious Diseases, Erasmus MC, Rotterdam, The Netherlands

**Keywords:** Malaria, Imported, Severity, Biomarkers, Copeptin, Arginine vasopressin, C-reactive protein, CRP, Traveller

## Abstract

**Background:**

Copeptin has recently been identified to be a stable surrogate marker for the unstable hormone arginine vasopressin (AVP). Copeptin has been shown to correlate with disease severity in leptospirosis and bacterial sepsis. Hyponatraemia is common in severe imported malaria and dysregulation of AVP release has been hypothesized as an underlying pathophysiological mechanism. The aim of the present study was to evaluate the performance of copeptin as a predictor of disease severity in imported malaria.

**Methods:**

Copeptin was measured in stored serum samples of 204 patients with imported malaria that were admitted to our Institute for Tropical Diseases in Rotterdam in the period 1999-2010. The occurrence of WHO defined severe malaria was the primary end-point. The diagnostic performance of copeptin was compared to that of previously evaluated biomarkers C-reactive protein, procalcitonin, lactate and sodium.

**Results:**

Of the 204 patients (141 *Plasmodium falciparum*, 63 non-falciparum infection), 25 had severe malaria. The Area Under the ROC curve of copeptin for severe disease (0.66 [95% confidence interval 0.59-0.72]) was comparable to that of lactate, sodium and procalcitonin. C-reactive protein (0.84 [95% CI 0.79-0.89]) had a significantly better performance as a biomarker for severe malaria than the other biomarkers.

**Conclusions:**

C-reactive protein but not copeptin was found to be an accurate predictor for disease severity in imported malaria. The applicability of copeptin as a marker for severe malaria in clinical practice is limited to exclusion of severe malaria.

## Background

Arginine vasopressin (AVP), also known as the antidiuretic hormone, is a key hormone in maintaining fluid balance and vascular tone. Despite its clinical relevance, measurement of mature AVP is difficult and subject to (pre)analytical errors. Recently, copeptin, a 39-amino acid glycopeptide that comprises the C-terminal part of the AVP precursor was found to be a stable and sensitive surrogate marker for AVP release [1,2]. In recent studies in patients with bacterial sepsis but also in patients with leptospirosis it was shown that copeptin levels correlate well with disease severity and outcome when compared to other commonly used biomarkers like C-reactive protein (CRP) and more experimental biomarkers like procalcitonin [3,4]. Copeptin levels may even be used as a valuable tool to guide management of critically ill patients [4]. Leptospirosis and bacterial sepsis are strikingly similar to severe malaria in terms of case-fatality rates and clinical presentation including the presence of hyponatraemia [5-9]. Hyponatraemia was found to be highly prevalent in severe malaria and suggested to be the result of a dysregulated vasopressin release [6-8].

The present study, therefore, aimed to evaluate copeptin as a pathophysiologic predictor of disease severity in patients with imported malaria. The diagnostic performance of copeptin was compared to that of several previously validated biomarkers to provide the clinician with the most accurate tool for clinical decision making in the acute care setting. A reliable test, easily discriminating severe from non-severe disease in malaria, would be clinically useful, especially in the setting of imported malaria, given the observation that most clinicians in non-endemic areas have little expertise with identifying severe malaria but these patients require a rapid triage for parenteral anti-malarials and intensive monitoring.

## Methods

The Harbour Hospital is a 161-bed general hospital located in Rotterdam, The Netherlands. It also harbours the Institute for Tropical Diseases, which serves as a national reference centre. In the period 1999-2010 more than 500 cases of imported malaria were diagnosed. The characteristics of the Rotterdam Malaria Cohort in the period 1999-2008 were previously described [9]. For the majority of these cases, demographic, clinical and laboratory data and serum samples were available. In the present study copeptin and procalcitonin were retrospectively measured in stored serum samples of 204 patients with imported malaria. The tested serum samples were not from consecutive patients entering this cohort but based on availability. The occurrence of severe malaria was considered a primary end-point given the low case-fatality rate of imported malaria in the Netherlands. Because severe malaria can also occur in non-falciparum infections (albeit rare in returning travellers), patients with non-falciparum infections were not excluded. This contrasts with the design of many studies in patients with severe malaria in regions of malaria endemicity where the severity criteria are usually used as an entry criterion [10] and focus on *P. falciparum *malaria. In the present study, the previously validated biomarkers plasma lactate [11], procalcitonin [12,13], sodium and CRP [9] were used as comparative biomarkers for copeptin to evaluate its accuracy for use in clinical practice to differentiate severe from non-severe malaria.

### Procedures

On admission, blood samples were taken for analysis of the red blood cell count, haematocrit, white blood cell count, platelet count, CRP, serum electrolytes, total bilirubin, serum creatinin, liver enzymes, and blood glucose. A separate blood tube was drawn on admission for determination of plasma lactate, which was immediately analysed after isolation of plasma. Malaria was diagnosed by QBC (Quantitative Buffy Coat) analysis, by a rapid diagnostic antigen test for malaria (Binax NOW^® ^Malaria Test, Binax Inc. Maine, USA) and by conventional microscopy of stained thick and thin blood smears to identify the causative *Plasmodium *species. In case of *Plasmodium falciparum *infections, parasite density was determined. When the parasitaemia was less than 0.5% infected erythrocytes, parasites were counted per 100 leucocytes in thick smears. When the parasitaemia was equal or higher than 0.5% infected erythrocytes, infected erythrocytes were counted in thin smear and expressed as a percentage of the total erythrocytes. The number of parasites per microliter was subsequently calculated from these data. Patients were classified as having severe malaria if they met one or more of the WHO criteria for severe malaria, as published [14].

Procalcitonin levels were determined in serum samples using a commercially available EIA test (VIDAS BRAHMS Procalcitonin, bioMérieux, Lyon, France). Normal serum values for procalcitonin are < 0.1 ng/ml. Copeptin was measured with a commercial sandwich immunoluminometric assay (Brahms Copeptin, Thermo Fisher Scientific, Hennigsdorf/Berlin, Germany). Normal values for serum copeptin are 1.70-11.25 pmol/L [2].

### Statistical methods

Differences between patients with severe malaria, non-severe *P. falciparum *malaria and non-severe non-falciparum malaria were analysed with the Kruskall-Wallis test followed by Dunn's post-hoc tests. Two group comparisons were done by Student's unpaired *t*-test, Student's unpaired *t*-test with Welch correction or with the non-parametric Mann Whitney test depending on the distribution of the data. The diagnostic performance of each biomarker was reported as sensitivity, specificity, positive and negative predictive value for severe malaria and their corresponding 95% confidence intervals. Youden's index J (J = sensitivity + specificity-1) was used to identify the most appropriate cut-off point for each biomarker. Of each test a Receiver Operating Characteristics (ROC) curve, a graphical plot of sensitivity (true positive rate) versus 1-specificity (false positive rate), was constructed as a summary statistic and the area under the ROC curve (AUROC) and its corresponding 95% confidence intervals were calculated. The AUROC of each of the biomarkers was compared to that of copeptin in a pair-wise comparison with the method of DeLong et al. [15].

## Results

### Patient characteristics

In total, 204 travellers with imported malaria were included in this study; 63 patients were diagnosed with a non-falciparum infection (45 with *Plasmodium vivax*, 15 with *Plasmodium ovale*, three with *Plasmodium malariae*) and 141 patients were diagnosed with *P. falciparum *infection. Of the patients with *P. falciparum *infections, 25 (17.7%) patients had severe malaria. There were no cases of severe non-falciparum malaria. The infection was most frequently acquired in Africa (n = 156), followed by Asia (n = 27) and South America (n = 14). Thirty (14.7%) patients reported adequate use of malaria chemoprophylaxis, 24 (11.8%) used it inadequately and 143 (70.1%) patients did not use any chemoprophylaxis (data on prophylaxis use were not available in seven patients). The general characteristics of all patients are shown in Table 1.

**Table 1:** Characteristics of malaria patients at initial presentation

	Severe malaria [all *P. falciparum*] (n = 25)	Non-severe *P. falciparum* (n = 116)	Non-severe Non-falciparum (n = 63)	*P*-value
**Demographics**				
Age, years	**44 (23-70)**	**41 (11-69)**	**38 (8-62)**	**n.s**.
Male, female, n (%)	**15 (60), 10 (40)**	**92 (79), 24 (21)**	**44 (70), 19 (30)**	**n.s**.
Duration of complaints, n (%)				**0.0905**
Less than 1 week	**16 (64)**	**83 (72)**	**31 (49)**	
1-2 weeks	**8 (32)**	**20 (17)**	**15 (24)**	
3-4 weeks	**0 (0)**	**8 (7)**	**5 (8)**	
More than 4 weeks	**0 (0)**	**1 (1)**	**3 (5)**	
**Vital signs on admission**				
Body temperature, °C	**38.4 (35.7-40.6)**	**38.6 (35.7-41.0)**	**38.3 (36.0-41.2)**	**n.s**.
Pulse rate, beats per minute	**108 (75-140)^A < 0,001; B < 0,01^**	**90 (68-130)**	**90 (58-138)**	**0.0005**
Systolic blood pressure, mm Hg	**118 (80-150)**	**120 (88-185)**	**120 (95-196)**	**n.s**.
**Laboratory data on admission**				
Parasite density (parasites/μL)	**284,005 (39,600-1,380,600)**	**22,657 (2-156,600)**	**n.a**.	**< 0.0001**
C-reactive protein, mg/L	**186 (71-407)^A < 0,001; B < 0,001^**	**95 (7-310)**	**83 (14-348)**	**< 0.0001**
Haemoglobin, mmol/L	**7.8 (2.5-10.2)^A < 0,05^**	**8.5 (5.3-11.1)**	**8.3 (5.6-10.7)**	**0.0281**
Haematocrit, L/L	**0.37 (0.12-0.50)^A < 0,05^**	**0.41 (0.24-0.52)**	**0.39 (0.26-0.53)**	**0.0067**
Leucocyte count, × 10^9^/L	**6.9 (2.5-18.5)^A < 0,05^**	**5.0 (2.2-12.6)**	**5.3 (1.9-11.0)**	**0.0361**
Thrombocytes, × 10^9^/L	**36 (11-164)^A < 0,001; B < 0,001^**	**93 (16-385)**	**95 (10-292)**	**0.0004**
Serum glucose, mmol/L	**6.8 (4.1-8.8)**	**6.8 (4.4-26.0)**	**6.4 (4.2-22.1)**	**n.s**.
Serum sodium, mmol/L	**131 (124-139)^A < 0.05;B < 0,01^**	**134 (122-141)^C < 0,05^**	**136 (127-141)**	**< 0.0001**
Serum creatinine, μmol/L	**110 (70-1081)^B < 0,001^**	**97 (51-208)^C < 0,05^**	**91 (46-126)**	**0.0010**
Serum urea, mmol/L	**7.6 (3.8-55.8)^A < 0,01; B < 0,01^**	**5.2 (2.2-18.7)**	**5.1 (2.7-10.9)**	**0.0018**
Plasma lactate, mmol/L	**2,3 (1,0-5,8)^A < 0,001; B < 0,05^**	**1,4 (0,5-5,5)**	**1.7 (0.7-4.0)**	**0.0014**
Procalcitonin, ng/mL	**1.9 (0.9-42.3)^A < 0.01^**	**0.6 (0.0-11.2)**	**1.6 (0.0-42.6)**	**0.0057**
Copeptin, pmol/L	**22.9 (5.1-91.5) ^B < 0,05^**	**13.9 (1.6-67.8)**	**12.0 (1.9-82.9)**	**0.0249**
**Duration hospitalisation**, days	**7 (3-13)^A < 0,001; B < 0,001^**	**5 (0-10)^C < 0,001^**	**2 (0-9)**	**< 0.0001**

Statistical analysis: Kruskall-Wallis (p-values in column) followed by Dunn's post-hoc tests (*p*-values in superscript); n.s. denotes "not significant"; n.a. denotes "not applicable". A: severe malaria *vs *non-severe *P. falciparum *malaria; B: severe malaria *vs *non-severe non-falciparum malaria; C: non-severe *P. falciparum *malaria *vs *non-severe non-falciparum malaria. Comparison of parasite density was calculated for *P. falciparum *infections only (Mann Whitney test)

### Characteristics of patients with severe malaria

Twenty-one of 25 patients (84.0%) with severe malaria acquired a *P. falciparum *infection in West-Africa. The others acquired severe malaria in East-Africa (n = 2), South-East Asia (n = 1) and South-America (n = 1). Twenty-four patients with severe malaria did not use any form of malaria chemoprophylaxis and one patient was using it inadequately. At admission to the intensive care unit (ICU), the 25 patients with severe malaria all fulfilled one or more of the severity criteria (Glasgow Coma Score ≤ 11, n = 2; haematocrit < 0.20 L/L n = 5; creatinine > 250 μmol/L n = 2; bilirubin > 50 μmol/L n = 12; lactate > 5.0 mmol/L n = 5; hyperparasitaemia > 5% n = 14; schizontaemia n = 14). The first arterial blood gas analysis on the ICU showed a median bicarbonate level of 21 mmol/L (range 9-25 mmol/L) and a median base excess of -2 mmol/L (range -18-3). The median Glasgow Coma Scale score was 15 (range 11-15). Two patients required mechanical ventilation. Two patients were referred to another hospital for renal replacement therapy because of persistent acute oliguric renal insufficiency. Thirteen patients received exchange transfusion as an adjunct therapy to parenteral anti-malarial treatment. One case fatality was observed. The laboratory results of travellers with severe malaria were further characterized by significantly lower platelet counts and haemoglobin levels and by significantly higher plasma lactate, bilirubin and CRP levels compared to patients with non-severe *P. falciparum *malaria (Table 1).

### Analysis of biomarkers for severe malaria

**Figure 1:**
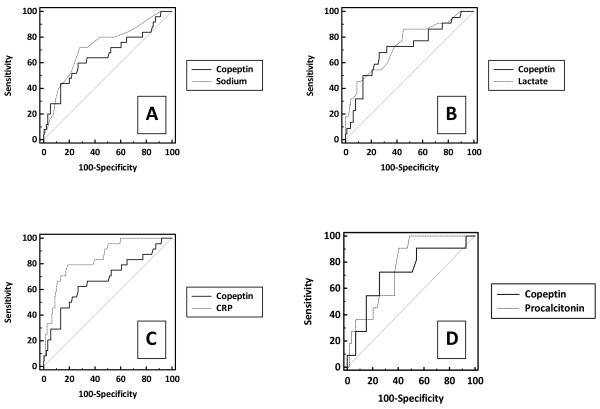
**Receiver Operating Curves (ROC) characteristics of the diagnostic performance of copeptin for severe *P. falciparum *malaria, compared to that of sodium, lactate, CRP and procalcitonin**. The ROC curve is a graph of sensitivity (true positive fraction) plotted against 1-specificity (false positive fraction). The performance of a diagnostic variable can be quantified by calculating the area under the ROC curve (AUROC). The ideal test would have an AUROC of 1, whereas a random guess would have an AUROC of 0.5. The results of the pair-wise comparison of ROC curves are shown (with copeptin ROC curve as comparator). **a**. copeptin vs sodium (n = 204 pairs, *p*-value not significant); **b**. copeptin vs lactate (n = 124 pairs, *p*-value not significant); **c**. copeptin vs CRP (n = 202 pairs, p = 0.02); **d**. copeptin vs procalcitonin (n = 70 pairs, *p*-value not significant). The copeptin graphs are not all identical due to missing values in pair-wise comparisons.

In order to evaluate the diagnostic accuracy of copeptin for severe malaria, the performance of copeptin was compared to that of sodium, CRP, lactate and procalcitonin. Assuming that AUROCs are a measure of diagnostic accuracy, the performance of copeptin for severe malaria was comparable to that of sodium, lactate and procalcitonin, as is shown in Figure 1. The AUROC of copeptin did not statistically differ from the AUROC of sodium, lactate and procalcitonin in a pair-wise comparison (Table 2). As shown in Figure 1 and Table 2, CRP had a significantly better performance than either copeptin, sodium and lactate for prediction of severe malaria but its usefulness in clinical practice is probably limited. For illustration, when a hypothetical decision rule for severe malaria was created solely based on a CRP cut-off level of ≥ 155 mg/L, 10 of 62 (or 16.1%) evaluable patients with non-falciparum disease and 26 of 116 (or 22.4%) evaluable patients with uncomplicated *P. falciparum *would have been falsely diagnosed as having severe malaria. In contrast, the diagnosis severe malaria would not have been considered in 5 of 24 (20.8%) evaluable patients with severe disease. Addition of either lactate, sodium or copeptin as a secondary selection criterion to the prediction rule did not result in a proper identification of all patients with severe disease on admission. Only after adding procalcitonin (at a cut-off of ≥ 0.9 ng/mL) as a secondary selection criterion to the prediction rule, all evaluable patients with severe malaria would have been correctly identified on admission (Additional file 1: Figure S1). However, still nine of 29 evaluable patients with non-severe *P. falciparum *malaria and 10 of 15 evaluable patients with non-severe, non-falciparum malaria would have been falsely diagnosed with severe malaria, respectively (Additional file 1: Figure S1).

**Table 2:** Descriptive statistics of diagnostic accuracy of the various biomarkers for severe malaria

	Copeptin (pmol/L)	Sodium (mmol/L)	Lactate (mmol/L)	C-reactive protein (mg/L)	Procalcitonin (ng/mL)
Cut-off value	^3 ^21	≤ 132	≥ 1.6	≥ 155	≥ 0.9

Youden's index	0.33	0.44	0.41	0.59	0.51

Sensitivity	60 (39-78)	72 (50-87)	86 (64-96)	79 (57-92)	100 (68-100)

Specificity	73 (66-79)	72 (64-78)	56 (45-65)	80 (73-85)	51 (38-64)

Positive Predictive Value	31 (25-38)	26 (0.17-0.38)	29 (19-42)	35 (23-49)	28 (15-44)

Negative Predictive Value	93 (87-96)	95 (89-98)	95 (85-99)	97 (92-99)	100 (86-100)

Area Under the ROC curve	0.66 (0.59-0.72)	0.72 (0.66-0.78)	0.74 (0.66-0.81)	0.84 (0.79-0.89)	0.76 (0.65-0.86)

*P*-value*		*P *= 0.4289	*P *= 0.6546	*P *= 0.0237	*P *= 0.6479

Data are given as mean (95% confidence interval)

*P-values of pair-wise comparison of ROC curves are given (with Copeptin ROC curve as comparator)

### Analysis of biomarkers in relation to the number of severity criteria in patients with severe disease

When focusing on the 25 patients with severe malaria, 11 patients fulfilled a single criterion for severe malaria on admission, 11 patients fulfilled two malaria severity criteria, 2 patients fulfilled 3 malaria severity criteria and one patient fulfilled 4 malaria severity criteria on admission to the ICU, respectively. As a consequence of this distribution a proper stratification by numbers of malaria severity criteria present on admission was not feasible. When the severe malaria patients fulfilling a single malaria severity criterion on admission were compared to the patients with more than one criterion for severe malaria on admission, only for the biomarker plasma lactate a statistically significant difference was found (1.8 ± 0.6 versus 3.3 ± 1.6 mmol/L, *p *= 0.0042). See Additional file 2: Table S1 for further details.

## Discussion

In the present study, the diagnostic performance of several biomarkers for severe malaria was evaluated. In contrast to studies in patients with severe bacterial sepsis [3] and severe leptospirosis [4], copeptin was not found to be a more accurate marker to predict severe disease in malaria than the previously validated biomarkers procalcitonin, sodium, and lactate. This may suggest that the major clinical manifestations of severe bacterial sepsis and leptospirosis may follow other pathophysiologic pathways than in patients with severe malaria. In the latter group of patients, CRP had a clearly superior performance as compared to the previously mentioned biomarkers including copeptin. This finding is certainly of clinical relevance since measurement of CRP protein is not only widely available in industrialized countries but also readily available during night shifts in the acute care setting.

The current findings implicate that a CRP level above 155 mg/l predicts severe malaria with a sensitivity and specificity of 79% and 80%, respectively. However, the usefulness of CRP for the prediction of severe malaria in clinical practice is probably limited. To illustrate this in more detail: when a hypothetical decision rule for severe malaria was constructed solely based on CRP and applied to the current study population, 16% of the evaluable patients with non-severe, non-falciparum malaria and 22% of the evaluable patients with non-severe *P. falciparum *malaria would have been falsely diagnosed as having severe malaria, whereas the diagnosis severe malaria would not have been considered in 21% of the evaluable patients with severe disease. Addition of either copeptin, sodium or lactate to the prediction rule did not result in a proper identification of all patients with severe malaria. Only adding procalcitonin ≥ 0.9 ng/mL as a secondary selection criterion to the prediction rule would have correctly identified all patients with severe malaria on admission at the price of overestimating the disease severity in a considerable number of patients with non-severe *P. falciparum *malaria and patients with non-severe, non-falciparum malaria, respectively. Applying this decision rule as a guide to therapy would, therefore, still lead to a significant proportion of malaria patients receiving more intensive monitoring and treatment than strictly necessary.

In addition, there may be another pitfall with CRP-based decision rules in malaria. As is known from studies in patients with severe malaria, co-infections may occur frequently in patients with malaria, which in itself may lead to a rise in CRP levels, irrespective of the malaria disease severity. For example, in a large cohort of 400 cases of imported severe malaria, 96 first episodes of co-infection were seen, in particular pneumonia (n = 61), bacteraemia (n = 18) and urinary tract infections (n = 12). Interestingly, these episodes were related to both community-acquired (n = 30) as well as nosocomial infections (n = 66) [16]. In our study blood cultures of patients with severe malaria did not demonstrate presence of co-existing bacteraemia on admission.

These abovementioned observations underline the limited value of these biomarkers for a rapid diagnosis of severe disease in the acute care setting in non-endemic industrialized countries; as for now, a biomarker-based decision rule can therefore certainly not replace the current clinical evaluation. It is noteworthy that all currently studied biomarkers were characterized by high negative predictive values, which may be helpful for exclusion of severe malaria on admission. The combined use of biomarkers may look promising since they allow correct identification of all patients with severe malaria but their use in the current clinical practice is hampered by their poor positive predictive values and lack of prospective validation.

In conclusion, copeptin was not found to be a good biomarker for severe malaria in imported malaria. Instead, CRP appeared to be a more accurate predictor for disease severity than the other investigated biomarkers. The main clinical applicability of the current biomarkers or combination of biomarkers is probably limited to a rapid exclusion of severe disease given their high negative predictive values and low positive predictive values.

## Competing interests

The authors declare that they have no competing interests.

## Authors' contributions

MEvW contributed to the data acquisition and analysis and writing of the manuscript. DAH, EJH and JvH participated in the data analysis and revising of the manuscript. YdeR carried out the copeptin measurements and contributed to the data analysis. RK is responsible for collection of patient materials and database management. PJvG participated in the data acquisition and analysis and in writing and revising the manuscript. All authors have read and approved the final version.

## Supplementary Material

Additional file 1 **Figure S1 A two-step decision rule including the combined use of C-reactive protein and Procalcitonin to identify all patients with severe malaria on admission**. Legend: SF = severe *P. falciparum *malaria; NSF = non-severe *P. falciparum *malaria; NSNF = non-severe, non-falciparum malaria; CRP = C-reactive protein; PCT = Procalcitonin. Click here for file

Additional file 2 **Table S1 Impact of the number of severity criteria on the level of the biomarker on admission in malaria patients with severe disease**. Click here for file

## References

[B1] Struck J Morgenthaler NG Bergmann A Copeptin, a stable peptide derived from the vasopressin precursor, is elevated in serum of sepsis patients Peptides 2005 26 2500 2504 10.1016/j.peptides.2005.04.019 15922490

[B2] Morgenthaler NG Struck J Alonso C Bergmann A Assay for the measurement of copeptin, a stable peptide derived from the precursor of vasopressin Clin Chem 2006 52 112 119 10.1373/clinchem.2005.060038 16269513

[B3] Jochberger S Morgenthaler NG Mayr VD Luckner G Wenzel V Ulmer H Schwarz S Hasibeder WR Friesenecker BE Dünser MW Copeptin and arginine vasopressin concentrations in critically ill patients J Clin Endocrinol Metab 2006 91 4381 4386 10.1210/jc.2005-2830 16940457

[B4] Limper M Goeijenbier M Wagenaar JF Gasem MH Isbandrio B Kunde J Hartmann O Duits AJ Van Gorp EC Copeptin as a predictor of disease severity and survival in leptospirosis J Infect 2010 6 92 94 10.1016/j.jinf.2010.03.029 20394769

[B5] Sitprija V Altered fluid, electrolyte and mineral status in tropical disease, with an emphasis on malaria and leptospirosis Nat Clin Pract Nephrol 2008 4 91 101 18227802 10.1038/ncpneph0695

[B6] Hanson J Hossain A Charunwatthana P Hassan MU Davis TM Lam SW Chubb SA Maude RJ Yunus EB Haque G White NJ Day NP Dondorp AM Hyponatremia in severe malaria: evidence for an appropriate antidiuretic hormone response to hypovolemia Am J Trop Med Hyg 2009 80 141 145 19141852 10.4269/ajtmh.2009.08-0393 PMC2843441

[B7] Fryatt RJ Teng JD Harries AD Moody AH Hall AP Forsling ML Plasma and urine electrolyte concentrations and vasopressin levels in patients admitted to hospital for falciparum malaria Trop Geogr Med 1989 41 57 60 2669290

[B8] Holst FG Hemmer CJ Kern P Dietrich M Inappropriate secretion of antidiuretic hormone and hyponatremia in severe falciparum malaria Am J Trop Med Hyg 1994 50 602 607 8203710 10.4269/ajtmh.1994.50.602

[B9] van Wolfswinkel ME Hesselink DA Zietse R Hoorn EJ van Genderen PJ Hyponatraemia in imported malaria is common and associated with disease severity Malar J 2010 25 140 10.1186/1475-2875-9-140 PMC2890675 20497587

[B10] Anstey NM Price RN Improving case definitions for severe malaria PLoS Med 2007 4 e267 10.1371/journal.pmed.0040267 17713984 PMC1949843

[B11] Van Genderen PJ van der Meer IM Consten J Petit PLC van Gool T Overbosch D Evaluation of plasma lactate as a parameter for disease severity on admission in travelers with *Plasmodium falciparum *malaria J Travel Med 2005 12 261 264 16256049 10.2310/7060.2005.12504

[B12] Hesselink DA Burgerhart JS Bosmans-Timmerarends H Petit P van Genderen PJ Procalcitonin as a biomarker for severe *Plasmodium falciparum *disease: a critical appraisal of a semi-quantitative point-of-care test in a cohort of travellers with imported malaria Malar J 2009 8 206 10.1186/1475-2875-8-206 19723338 PMC3224901

[B13] te Witt R van Wolfswinkel ME Petit PL van Hellemond JJ Koelewijn R van Belkum A van Genderen PJ Neopterin and procalcitonin are suitable biomarkers for exclusion of severe *Plasmodium falciparum *disease at the initial clinical assessment of travellers with imported malaria Malar J 2010 9 255 10.1186/1475-2875-9-255 20840738 PMC2946356

[B14] Guidelines for the treatment of malaria 2010 Second World Health Organization http://www.who.int/malaria/publications/atoz/9789241547925/en/index.html

[B15] DeLong ER DeLong DM Clarke-Pearson DL Comparing the areas under two or more correlated receiver operating characteristic curves: a nonparametric approach Biometrics 1988 44 837 845 10.2307/2531595 3203132

[B16] Bruneel F Tubach F Corne P Megarbane B Mira JP Peytel E Camus C Schortgen F Azoulay E Cohen Y Georges H Meybeck A Hyvernat H Trouillet JL Frenoy E Nicolet L Roy C Durand R Le Bras J Wolff M Severe imported falciparum malaria: a cohort study in 40 critically ill adults PLoS One 2010 5 e13236 10.1371/journal.pone.0013236 20949045 PMC2951913

