# Supplementary figures and images for: Copeptin does not accurately predict disease severity in imported malaria
Source: Malar J. 2012 Jan 5;11:6. doi: 10.1186/1475-2875-11-6 (PMC3268091; doi:10.1186/1475-2875-11-6)

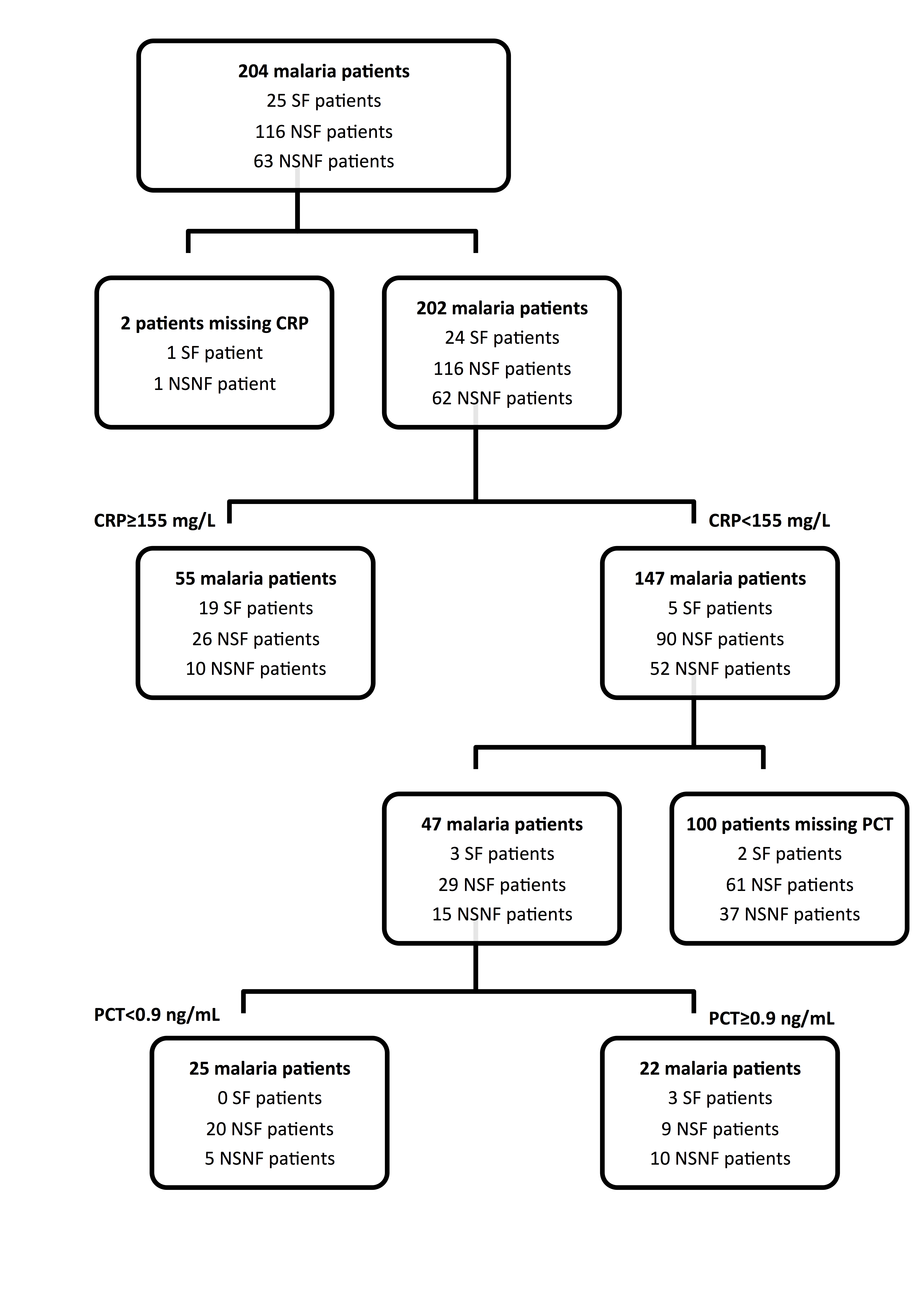

Supplement: Additional file 1 — Figure S1 A two-step decision rule including the combined use of C-reactive protein and Procalcitonin to identify all patients with severe malaria on admission. Legend: SF = severe P. falciparum malaria; NSF = non-severe P. falciparum malaria; NSNF = non-severe, non-falciparum malaria; CRP = C-reactive protein; PCT = Procalcitonin. [file 1475-2875-11-6-S1.JPEG]
