# Supplementary material for: Copeptin does not accurately predict disease severity in imported malaria
Source: Malar J. 2012 Jan 5;11:6. doi: 10.1186/1475-2875-11-6 (PMC3268091; doi:10.1186/1475-2875-11-6)
Supplement: Additional file 2 — Table S1 Impact of the number of severity criteria on the level of the biomarker on admission in malaria patients with severe disease. [file 1475-2875-11-6-S2.DOC]

| **Supplementary File 1**. Impact of the number of severity criteria on the level of the biomarker of interest on admission in malaria patients with severe disease | | | |
| --- | --- | --- | --- |
| Parameter | Patients with a single criterion for severe disease  (N=11) | Patients with more than one criterion for severe disease  (N=14) | P-value |
| C-reactive protein (mg/L) | 161 (111-210) | 225 (177-272) | P=0.058 |
| Sodium  (mmol/L) | 133 (130-135) | 131 (128-133) | P=0.349 |
| Lactate  (mmol/L) | 1.8 (1.0-2.7) | 3.3 (2.4-4.3) | P=0.004 |
| Copeptin  (pmol/L) | 25.4 (13.1-37.7) | 30.5 (15.7-45.2) | P=0.687 |
| Procalcitonin (ng/mL) | 4.1 (-2.3-10.6) | 17.4 (-7.1-42.0) | P=0.360 |
